# Pioglitazone and breast cancer risk in female patients with type 2 diabetes mellitus: a retrospective cohort analysis

**DOI:** 10.1186/s12885-022-09660-8

**Published:** 2022-05-18

**Authors:** Chin-Hsiao Tseng

**Affiliations:** 1 Department of Internal Medicine, National Taiwan University College of Medicine, No. 7 Chung-Shan South Road, Taipei, Taiwan; 2 Division of Endocrinology and Metabolism, Department of Internal Medicine, National Taiwan University Hospital, Taipei, Taiwan; 3 Division of Environmental Health and Occupational Medicine of the National Health Research Institutes, Zhunan, Taiwan

**Keywords:** Breast cancer, Diabetes mellitus, Pioglitazone, Taiwan

## Abstract

**Background:**

Whether pioglitazone may affect breast cancer risk in female diabetes patients is not conclusive and has not been investigated in the Asian populations.

**Methods:**

The reimbursement database of Taiwan’s National Health Insurance was used to enroll an unmatched cohort and a propensity score-matched cohort of ever users and never users of pioglitazone in female patients with newly diagnosed type 2 diabetes during 1999–2008. The patients were alive on January 1, 2009 and were followed up for breast cancer incidence until December 31, 2011. Cox regression was used to estimate hazard ratios for ever users and tertiles of cumulative duration of pioglitazone therapy versus never users, and for cumulative duration of pioglitazone therapy treated as a continuous variable. Three models were created for the unmatched cohort and the matched cohort, respectively: 1) without adjustment for covariates; 2) after adjustment for covariates that differed with statistical significance (*P*-value < 0.05) between ever users and never users; and 3) after adjustment for all covariates.

**Results:**

There were 174,233 never users and 6926 ever users in the unmatched cohort; and 6926 never users and 6926 ever users in the matched cohort. After a median follow-up of 2.8 years, the numbers of incident breast cancer were 1044 in never users and 35 in ever users in the unmatched cohort and were 41 and 35, respectively, in the matched cohort. Hazard ratios suggested a null association between pioglitazone and breast cancer in all three models in either the unmatched cohort or the matched cohort. The overall hazard ratio after adjustment for all covariates was 0.758 (95% confidence interval: 0.539–1.065) in the unmatched cohort and was 0.824 (95% confidence interval: 0.524–1.296) in the matched cohort. None of the hazard ratios for the tertiles of cumulative duration of pioglitazone therapy and for the cumulative duration being treated as a continuous variable were statistically significant.

**Conclusions:**

This study suggests a null association between pioglitazone and breast cancer risk in female patients with type 2 diabetes mellitus. However, because of the small breast cancer cases and the limited follow-up time, further studies are warranted to confirm our findings.

## Introduction

The safety monitoring data of several previous clinical trials that compared the risk of cardiovascular disease between pioglitazone and placebo [1, 2] or between pioglitazone and sulfonylurea on top of metformin [3] have shown lower case numbers of incident breast cancer in patients randomized to pioglitazone than to comparators (3:11 [1], 10:16 [2] and 3:4 [3]). However, these clinical trials were not designed primarily for investigating breast cancer risk as an endpoint and therefore the small numbers of incident cases of breast cancer in the safety monitoring data indicated a lack of sufficient power.

There are several pharmacoepidemiological studies, all conducted in Caucasians, investigating breast cancer risk associated with use of pioglitazone and/or rosiglitazone. Analyses of the US Kaiser Permanente Northern California (KPNC, interim analysis) Diabetes Registry [4] and the French national health insurance database showed a null association between pioglitazone and female breast cancer, though the French study did find a lower risk of breast cancer associated with rosiglitazone with statistical significance [5]. However, Lewis et al. showed, in the final report of the KPNC data, that an increasing trend of breast cancer could be observed with increasing dose and duration of pioglitazone in sensitivity analyses [6]. Therefore, results from clinical trials and pharmacoepidemiological studies conducted in Caucasians showed contradictory findings.

In Taiwan, previous pharmacoepidemiological studies suggested that metformin [7] and rosiglitazone [8], both improve insulin resistance, may lower the risk of breast cancer. Therefore, it would be interesting to further examine whether pioglitazone, another insulin sensitizer, might also have a beneficial effect on breast cancer in the Asian populations. The present study investigated such an association in female patients with type 2 diabetes mellitus in Taiwan by using the reimbursement database of the National Health Insurance (NHI).

## Materials and methods

This is a retrospective cohort analysis of Taiwan’s NHI reimbursement database. The NHI, a compulsory and universal healthcare system in Taiwan, has been implemented since March 1995. More than 99% of the population are covered by the NHI, and all in-hospitals and 93% of all medical settings have contracts with the NHI. The NHI database contains detailed records of every visit of each patient and includes principal and secondary diagnostic codes, prescription orders and procedures performed. The present study was approved after ethics review by the National Health Research Institutes with number 99274. Informed consent was not required according to local regulations because the database has been de-identified for the protection of privacy.

During the study period diabetes was coded 250.XX and breast cancer 174, based on the *International Classification of Diseases, Ninth Revision, Clinical Modification* (ICD-9-CM).

**Fig. 1:**
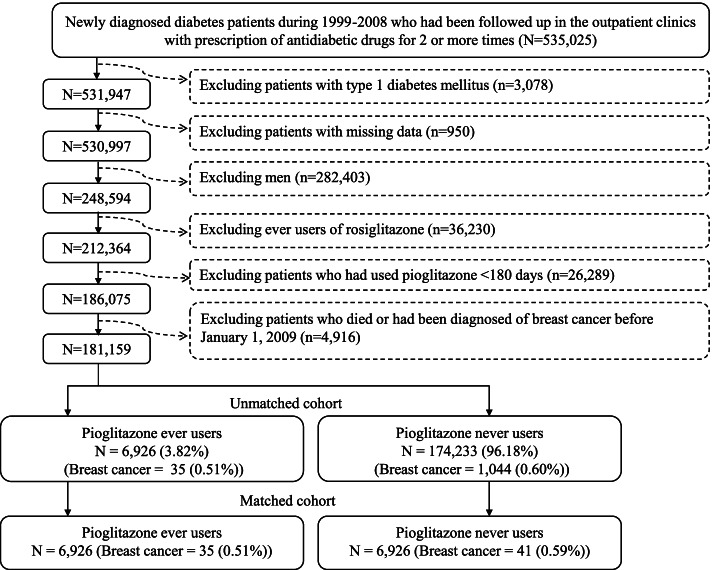
Flowchart showing the procedures in creating a cohort of 1:1 matched-pairs of pioglitazone ever and never users from the reimbursement database of the National Health Insurance

More detailed description of the database can be seen in previously published papers [9, 10]. Figure 1 shows the procedures in enrolling an unmatched cohort and a matched cohort of pioglitazone ever users and never users based on propensity score. Patients with newly diagnosed diabetes during 1999–2008 in the outpatient clinics and having been prescribed antidiabetic drugs for 2 or more times were first identified (*n* = 535,025). To ensure a newly diagnosed diabetes after 1999, patients having a diagnosis of diabetes between 1996 and 1998 were not included. The following patients were then excluded: 1) type 1 diabetes mellitus (*n* = 3078), 2) missing data (*n* = 950), 3) men (*n* = 282,403), 4) use of rosiglitazone (*n* = 36,230, users of rosiglitazone were excluded because previous *in vitro* and *in vivo* [11] and human observational [6] studies suggested that rosiglitazone may act differently from pioglitazone in breast cancer), 5) pioglitazone use for < 180 days (*n* = 26,289) and 6) patients who died or had been diagnosed of breast cancer before January 1, 2009 (*n* = 4916). As a result, 6926 ever users and 174,233 never users of pioglitazone were identified as the unmatched cohort. A cohort of 6926 ever users and 6926 never users of pioglitazone (the matched cohort) was created by matching the propensity score based on the Greedy 8➔1 digit match algorithm [12]. Logistic regression was used to create the propensity score from all characteristics listed in Table 1. This matching method has been described in more detail elsewhere [9, 10].

**Table 1:** Characteristics in never and ever users of pioglitazone in the unmatched cohort and the propensity score-matched cohort

Variable	Unmatched cohort						Matched cohort					
	Never users		Ever users				Never users		Ever users			
	(*n* = 174,233)		(*n* = 6926)		*P* value	SD	(*n* = 6926)		(*n* = 6926)		*P* value	SD
	n	%	n	%			n	%	n	%		
**Demographic data**												
Age (years)	63.21	12.53	60.12	10.93	< 0.0001	−30.19	60.44	12.36	60.12	10.93	0.1074	−2.91
Diabetes duration (years)	5.30	2.90	6.75	2.56	< 0.0001	55.74	6.81	2.77	6.75	2.56	0.2468	−2.15
Occupation												
I	56,692	32.54	2406	34.74	< 0.0001		2450	35.37	2406	34.74	0.0987	
II	39,108	22.45	1805	26.06		6.70	1701	24.56	1805	26.06		3.39
III	43,636	25.04	1416	20.44		−16.08	1502	21.69	1416	20.44		−3.10
IV	34,797	19.97	1299	18.76		2.11	1273	18.38	1299	18.76		0.94
Living region												
Taipei	56,595	32.48	2711	39.14	< 0.0001		2689	38.82	2711	39.14	0.9897	
Northern	22,911	13.15	716	10.34		−7.30	720	10.40	716	10.34		−0.17
Central	29,751	17.08	1142	16.49		2.12	1139	16.45	1142	16.49		0.03
Southern	30,602	17.56	873	12.60		−21.02	870	12.56	873	12.60		0.23
Kao-Ping and Eastern	34,374	19.73	1484	21.43		4.32	1508	21.77	1484	21.43		−0.91
**Major comorbidities**												
Hypertension	137,813	79.10	5486	79.21	0.8224	−1.76	5552	80.16	5486	79.21	0.1634	−2.40
Dyslipidemia	128,253	73.61	5633	81.33	< 0.0001	21.24	5568	80.39	5633	81.33	0.1603	2.41
Obesity	10,282	5.90	455	6.57	0.0209	1.40	499	7.20	455	6.57	0.1399	−2.48
**Diabetes-related complications**												
Nephropathy	35,634	20.45	1313	18.96	0.0025	−6.46	1319	19.04	1313	18.96	0.8966	−0.23
Eye disease	25,172	14.45	1942	28.04	< 0.0001	27.93	1929	27.85	1942	28.04	0.8056	0.39
Stroke	48,431	27.80	1552	22.41	< 0.0001	−16.40	1544	22.29	1552	22.41	0.8704	0.16
Ischemic heart disease	77,411	44.43	2746	39.65	< 0.0001	−10.82	2808	40.54	2746	39.65	0.2824	−1.89
Peripheral arterial disease	35,540	20.40	1473	21.27	0.0783	0.08	1478	21.34	1473	21.27	0.9174	−0.35
**Antidiabetic drugs**												
Insulin	6662	3.82	184	2.66	< 0.0001	−18.45	178	2.57	184	2.66	0.7493	0.46
Sulfonylurea	99,885	57.33	4780	69.02	< 0.0001	19.40	4812	69.48	4780	69.02	0.5557	−1.15
Metformin	120,594	69.21	5022	72.51	< 0.0001	−11.46	4995	72.12	5022	72.51	0.6082	0.92
Meglitinide	8434	4.84	466	6.73	< 0.0001	5.45	441	6.37	466	6.73	0.3905	1.35
Acarbose	12,820	7.36	916	13.23	< 0.0001	11.30	906	13.08	916	13.23	0.8015	0.35
**Factors that may affect cancer risk or lifespan**												
Chronic obstructive pulmonary disease	85,452	49.04	3013	43.50	< 0.0001	−14.74	3070	44.33	3013	43.50	0.3291	−1.61
Tobacco abuse	1034	0.59	37	0.53	0.5282	−1.05	55	0.79	37	0.53	0.0597	−3.68
Alcohol-related diagnoses	4496	2.58	146	2.11	0.0147	−2.00	139	2.01	146	2.11	0.6752	0.66
Hypoglycemia	3803	2.18	157	2.27	0.6387	0.94	179	2.58	157	2.27	0.2244	−2.15
Head injury	3935	2.26	155	2.24	0.9102	−4.02	122	1.76	155	2.24	0.0452	3.23
Parkinson’s disease	5702	3.27	128	1.85	< 0.0001	−9.07	129	1.86	128	1.85	0.9498	−0.06
Benign breast conditions	34,506	19.80	1319	19.04	0.1192	0.82	1282	18.51	1319	19.04	0.4208	1.48
Cancers other than breast cancer prior to baseline	19,146	10.99	614	8.87	< 0.0001	−8.16	645	9.31	614	8.87	0.3595	−1.47
Potential detection bias	85,454	49.05	3093	44.66	< 0.0001	−9.97	3018	43.57	3093	44.66	0.1994	2.28
**Medications that are commonly used in diabetes patients or may affect cancer risk**												
Angiotensin converting enzyme inhibitor/angiotensin receptor blocker	114,565	65.75	4878	70.43	< 0.0001	10.05	4949	71.46	4878	70.43	0.1840	−2.12
Calcium channel blocker	107,812	61.88	4036	58.27	< 0.0001	−10.19	4144	59.83	4036	58.27	0.0620	−3.25
Statin	91,883	52.74	4694	67.77	< 0.0001	34.08	4709	67.99	4694	67.77	0.7849	−0.43
Fibrate	54,451	31.25	2593	37.44	< 0.0001	13.21	2583	37.29	2593	37.44	0.8606	0.24
Aspirin	95,675	54.91	3737	53.96	0.1169	−5.02	3767	54.39	3737	53.96	0.6089	−0.96
Estrogen	86,135	49.44	3422	49.41	0.9627	3.49	3427	49.48	3422	49.41	0.9323	0.08

*SD* Standardized difference

Age and diabetes duration are expressed as mean and standard deviation

Cumulative duration of pioglitazone therapy in months was calculated from the database and its tertiles were used to evaluate a possible dose-response relationship. Potential confounders included in the analyses were classified into the following categories. Demographic data included age, diabetes duration, occupation and living region (classified as Taipei, Northern, Central, Southern, and Kao-Ping/Eastern). Occupation was classified as class I (civil servants, teachers, employees of governmental or private businesses, professionals and technicians), class II (people without a specific employer, self-employed people or seamen), class III (farmers or fishermen) and class IV (low-income families supported by social welfare, or veterans). Major comorbidities included hypertension (ICD-9-CM: 401–405), dyslipidemia (272.0–272.4) and obesity (278). Diabetes-related complications included nephropathy (580–589), eye diseases (250.5, diabetes with ophthalmic manifestations, 362.0: diabetic retinopathy, 369: blindness and low vision, 366.41: diabetic cataract, and 365.44: glaucoma associated with systemic syndromes), stroke (430–438), ischemic heart disease (410–414) and peripheral arterial disease (250.7, 785.4, 443.81 and 440–448). Antidiabetic drugs included insulin, sulfonylureas, metformin, meglitinide and acarbose. Factors that may affect cancer risk or lifespan included chronic obstructive pulmonary disease (a surrogate for smoking; 490–496), tobacco abuse (305.1, 649.0 and 989.84), alcohol-related diagnoses (291, 303, 535.3, 571.0–571.3 and 980.0), hypoglycemia (251.0, 251.1 and 251.2), head injury (959.01), Parkinson’s disease (332), benign breast conditions (217, 610, 611, 612, 675 and 676) and cancers other than breast cancer prior to baseline (140–208, excluding 174). Some examinations that might potentially lead to the diagnosis of breast cancer were considered as an indicator of “potential detection bias”. These included 1) mammogram and/or breast ultrasound; 2) chest computed tomography and/or magnetic resonance imaging; and 3) tumor markers, including carcinoembryonic antigen and/or carbohydrate antigen 153. Commonly used medications in diabetes patients that may affect cancer risk included angiotensin converting enzyme inhibitor/angiotensin receptor blocker, calcium channel blocker, statin, fibrate, aspirin and estrogen.

Analyses were conducted in the unmatched cohort and the matched cohort, respectively. Student’s t test compared the difference of age and diabetes duration between never and ever users of pioglitazone and Chi-square test was used for other variables. Standardized difference was calculated for each variable and a value > 10% is considered as an indicator of potential confounding from the variable [13].

Incidence density of breast cancer was calculated with regards to the use of pioglitazone in the following subgroups: never users, ever users and the tertiles of cumulative duration. The case number of newly diagnosed breast cancer identified during follow-up was the numerator. The denominator was the follow-up duration in person-years, which started on January 1, 2009 and ended on December 31, 2011, at the time of a new diagnosis of breast cancer, or on the date of death or the last reimbursement record, whichever occurred first.

Hazard ratios and their 95% confidence intervals for ever users and for each tertile of cumulative duration in referent to never users were estimated by Cox proportional hazards model. Additionally, cumulative duration of pioglitazone therapy was treated as a continuous variable for estimating the hazard ratio. To examine the consistency of the findings, models were created in both the unmatched cohort and the matched cohort, respectively; and without adjustment for covariates, after adjustment for covariates with *P*-values < 0.05 and after adjustment for all covariates, respectively.

More antidiabetic drugs have been introduced into clinical practice and the guidelines for the use of antidiabetic drugs have evolved over the long enrollment period from 1999 to 2008. To examine whether the risk of breast cancer associated with pioglitazone use might change during different period of time, the overall hazard ratios were additionally estimated for two periods of time: 1999–2003 and 2004–2008, respectively.

Analyses were conducted using SAS statistical software, version 9.4 (SAS Institute, Cary, NC). *P* < 0.05 was considered statistically significant.

## Results

Table 1 shows the characteristics in never users and ever users of pioglitazone in the unmatched cohort and the matched cohort, respectively. In the unmatched cohort, most variables were statistically different between ever users and never users and the values of standardized difference were > 10% in many of the covariates, suggesting a potential confounding. However, in the matched cohort, except for head injury, all covariates did not differ significantly between the two groups and the values of standardized difference for all covariates were < 10%, indicating a good balance in all covariates between ever users and never users in the matched cohort.

Table 2 shows the incidences of breast cancer and hazard ratios by pioglitazone exposure estimated from different models in both the unmatched cohort and the matched cohort. The median follow-up time was 2.8 years in all subgroups. The incidence rates in never users and ever users were 239.83 and 191.90 per 100,000 person-years, respectively, in the unmatched cohort; and were 233.84 and 191.90 per 100,000 person-years, respectively, in the matched cohort. The hazard ratios suggested a null association between pioglitazone use and breast cancer in all models.

**Table 2:** Incidence rates of breast cancer and hazard ratios by pioglitazone exposure

Pioglitazone use	Incident case number of breast cancer	Cases followed	Person-years	Incidence rate (per 100,000 person-years)	Median follow-up (years)	Model 1			Model 2			Model 3		
						HR	95% CI	*P* value	HR	95% CI	*P* value	HR	95% CI	*P* value
**Unmatched cohort**														
Never users	1044	174,233	435,316.89	239.83	2.82	1.000			1.000			1.000		
Ever users	35	6926	18,238.48	191.90	2.84	0.800	(0.571–1.120)	0.1940	0.743	(0.528–1.044)	0.0867	0.758	(0.539–1.065)	0.1107
**Tertiles of cumulative duration of pioglitazone therapy (months)**														
Never users	1044	174,233	435,316.89	239.83	2.82	1.000			1.000			1.000		
< 10.67	9	2286	5854.05	153.74	2.82	0.643	(0.334–1.238)	0.1866	0.604	(0.313–1.165)	0.1325	0.620	(0.321–1.197)	0.1546
10.67–19.13	11	2278	5996.14	183.45	2.85	0.765	(0.422–1.385)	0.3761	0.703	(0.387–1.276)	0.2462	0.716	(0.394–1.299)	0.2712
> 19.13	15	2362	6388.28	234.81	2.84	0.978	(0.588–1.629)	0.9333	0.907	(0.543–1.515)	0.7079	0.922	(0.552–1.540)	0.7555
**Cumulative duration of pioglitazone treated as a continuous variable**														
For every 1-month increment of pioglitazone use						0.993	(0.978–1.008)	0.3395	0.990	(0.975–1.005)	0.1895	0.991	(0.976–1.006)	0.2197
**Matched cohort**														
Never users	41	6926	17,533.02	233.84	2.83	1.000			1.000			1.000		
Ever users	35	6926	18,238.48	191.90	2.84	0.818	(0.521–1.285)	0.3837	0.817	(0.520–1.282)	0.3787	0.824	(0.524–1.296)	0.4026
**Tertiles of cumulative duration of pioglitazone therapy (months)**														
Never users	41	6926	17,533.02	233.84	2.83	1.000			1.000			1.000		
< 10.67	9	2286	5854.05	153.74	2.82	0.658	(0.320–1.354)	0.2560	0.657	(0.319–1.351)	0.2534	0.655	(0.318–1.352)	0.2529
10.67–19.13	11	2278	5996.14	183.45	2.85	0.781	(0.402–1.520)	0.4671	0.779	(0.401–1.516)	0.4630	0.767	(0.394–1.495)	0.4357
> 19.13	15	2362	6388.28	234.81	2.84	0.999	(0.553–1.805)	0.9968	0.997	(0.552–1.802)	0.9925	1.043	(0.574–1.895)	0.8900
**Cumulative duration of pioglitazone treated as a continuous variable**														
For every 1-month increment of pioglitazone use						0.996	(0.978–1.013)	0.6202	0.996	(0.978–1.013)	0.6174	0.996	(0.979–1.014)	0.6841

Model 1: unadjusted; Model 2: adjusted for covariates in Table 1 with significant *P*-values; Model 3: adjusted for all covariates in Table 1

*HR* Hazard ratio, *CI* Confidence interval

Table 3 shows the overall hazard ratios for ever versus never users during two different periods of time. None of them suggested an effect of pioglitazone on breast cancer.

**Table 3:** Hazard ratios for breast cancer associated with pioglitazone use in patients with diabetes diagnosed in two different periods of time

	Unmatched cohort							Matched cohort						
Years diabetes diagnosed/Model	Ever users		Never users					Ever users		Never users				
	*n*	*N*	*n*	*N*	HR	95% CI	*P* value	*n*	*N*	*n*	*N*	HR	95% CI	*P* value
**1999–2003**														
Model 1	23	4632	449	74,459	0.790	(0.520–1.201)	0.2707	23	4632	25	4522	0.870	(0.494–1.533)	0.6295
Model 2	23	4632	449	74,459	0.694	(0.455–1.069)	0.0905	23	4632	25	4522	0.865	(0.491–1.524)	0.6148
Model 3	23	4632	449	74,459	0.709	(0.464–1.082)	0.1105	23	4632	25	4522	0.840	(0.475–1.488)	0.5508
**2004–2008**														
Model 1	12	2294	595	99,774	0.822	(0.464–1.455)	0.5005	12	2294	16	2404	0.736	(0.348–1.556)	0.4226
Model 2	12	2294	595	99,774	0.794	(0.447–1.411)	0.4319	12	2294	16	2404	0.738	(0.349–1.559)	0.4255
Model 3	12	2294	595	99,774	0.813	(0.457–1.445)	0.4808	12	2294	16	2404	0.740	(0.343–1.594)	0.4417

Model 1: unadjusted; Model 2: adjusted for covariates in Table 1 with significant *P*-values; Model 3: adjusted for all covariates in Table 1

## Discussion

This is the first observational study conducted in an Asian population that suggested a null association between pioglitazone use and breast cancer risk. The findings were consistent in the unmatched and the matched cohorts and in all models with different sets of adjusted covariates (Table 2). The finding of a null association was similarly observed in analyses conducted in patients whose diabetes was diagnosed during two different periods of time, i.e., 1999–2003 and 2004–2008 (Table 3).

Insulin resistance is an early pathophysiological change related to type 2 diabetes mellitus [14] and patients with type 2 diabetes mellitus are at an increased risk of breast cancer [15, 16]. Studies suggest that insulin resistance and hyperinsulinemia are important in the development of breast cancer [17, 18]. Therefore, it is hypothetically possible that breast cancer risk may be reduced by using antidiabetic drugs that improve insulin resistance. Our previous studies did show a reduction of breast cancer risk in patients who used either metformin [7] or rosiglitazone [8]. However, this study did not support a beneficial effect of pioglitazone, another antidiabetic drug that also improves insulin resistance, on breast cancer risk. The discrepant findings between pioglitazone and other insulin sensitizers including metformin and rosiglitazone suggest that factors other than the improvement of insulin resistance might be responsible.

Findings from some *in vitro* and *in vivo* studies may provide evidence to support these discrepant clinical observations. In a breast cancer cell line, rosiglitazone stimulates the expression of tumor suppressor gene *PTEN* (phosphatase and tensin homolog, located on chromosome ten) but pioglitazone does not exert a similar effect [11]. Another study showed that rosiglitazone exerts anti-proliferative and apoptotic actions on breast cancer cells; and induces autophagy and inhibits the invasiveness and metastasis of breast cancer cell lines [19]. In an animal study, rosiglitazone suppresses mammary tumor growth in rats treated with the carcinogen 7,12-dimethylbenz(a)anthracene [20]. On the other hand, although pioglitazone inhibits aromatase expression by inhibiting proinflammatory prostaglandin E2 signaling and upregulating tumor-suppressor gene BRCA1 [21], it does not inhibit mammary tumor growth induced by N-methyl-N-nitrosourea in Sprague-Dawley rats fed a high-fat diet [22]. Studies also suggested that metformin and pioglitazone might have different effects on breast cancer cells. A Turkish study showed that diabetes patients with breast cancer treated with metformin had statistically significant reduction of serum level of hypoxia-inducible factor-1α (a nuclear transcription factor overexpressed in breast cancer cells and correlated with cancer metastasis and mortality), but the level did not change after treatment with pioglitazone [23]. Taken together, these observations argued against a mechanism of breast cancer risk reduction associated with metformin and rosiglitazone merely through an improvement of insulin resistance and suggested that some other mechanisms might have traded off the beneficial effect of improvement in insulin resistance associated with pioglitazone.

After the withdrawal of rosiglitazone from the market because of a potential risk of macrovascular disease [24], pioglitazone is the only drug in the class of thiazolidinediones that remains in clinical use in most countries including Taiwan. The clinical trial (PROspective pioglitAzone Clinical Trial In macroVascular Events or the PROactive trial) published in 2005 that investigated the risk of cardiovascular disease comparing pioglitazone to placebo suggested a potentially higher risk of bladder cancer associated with pioglitazone use [1]. This has raised a concern of cancer risk associated with pioglitazone use and an observational prospective follow-up study (i.e., the KPNC study) was requested by the US Food and Drug Administration to clarify the risk of cancer, especially bladder cancer. The interim analyses of the KPNC study suggested a potentially higher risk of bladder cancer in patients who had been exposed to pioglitazone for a long duration or a high cumulative dose [25] but a null association with female breast cancer [4]. However, in the final report of the KPNC data, Lewis et al. showed weak linear trends in the risk of breast cancer associated with increasing cumulative dose and duration of pioglitazone use [6].

In the present study, we aimed at clarifying the effect of pioglitazone on breast cancer and therefore the balance of other potential confounders including the use of other antidiabetic drugs is important for an unbiased estimate. Although ever users and never users of pioglitazone differed significantly in the distribution of potential confounders in the unmatched cohort, they were well balanced in the matched cohort (Table 1). Because the results of a null association were consistent in different models in both the unmatched cohort and the matched cohort (Table 2), the conclusion should be robust and not affected by potential confounders.

We did not simultaneously investigate the effects of other antidiabetic drugs because no other antidiabetic drugs (except rosiglitazone that has been withdrawn from the market in many countries) had ever experienced such a great public health concern. The restrictions imposed by regulatory authorities after the publication of the interim analysis of the KPNC in 2011 [25] on the use of pioglitazone because of its potential risk of bladder cancer have caused tremendous psychological impacts not only to the physicians who would be reluctant to prescribe the drug but also to the patients who might not have adhered to taking the drug even when they had been prescribed pioglitazone. Therefore, the time frame to be considered in study design for an investigation on pioglitazone effect should be cautious and would surely be different as for other antidiabetic drugs.

The patients were enrolled from 1999 to 2005 and followed up until 2011. This database seemed to be too old. However, the study period was deliberately selected to reduce potential biases based on the following considerations. First, this time frame would avoid unidentifiable biases resulting from the impacts of the publication of the interim analysis of the KPNC study in 2011 [25] and the restriction of pioglitazone use imposed by regulatory authorities since then. Second, the Bureau of the NHI started to promote the use of ICD-10-CM in Taiwan since 2012 and therefore a potential bias resulting from a mixture of two disease coding systems might have happened if the follow-up ended after 2012.

It was also deemed inappropriate to investigate too many drugs and too many different cancers in a single study especially when pioglitazone was the target drug to be investigated because of the following reasons. First, as previously mentioned, the time frame for studying pioglitazone should be carefully restricted so that the findings would not be biased. The restriction on the use of pioglitazone would also affect the prescription and the adherence of other antidiabetic drugs and these behavior changes might have caused unexpected biases. Second, different antidiabetic drugs have different indications, contraindications and side effects and different cancers have different risk factors. It would be complicated to balance different sets of confounders. Third, cancer screening programs are evolving and different for different cancers. It may not be possible to simultaneously consider the impacts of these different screening programs when too many cancers are investigated in one single study.

Breast cancer screening programs have been conducted in either the USA [26] or in France [27] throughout the study periods of the clinical trials [1–3] and the observational study of the KPNC conducted in the USA [4, 6] and the observational study conducted in France [5]. These breast cancer screening programs can lead to detection bias. However, none of the early studies investigating the risk of breast cancer associated with pioglitazone use have addressed the potential impacts of breast cancer screening programs.

In Taiwan, breast cancer screening programs have evolved from hospital-based project (1995–1998), to community-based projects (1999–2001 and 2002–2004) and finally to nationwide programs (phase I since July 2004, phase II since November 2009 and phase III since 2010) [28]. The phase I nationwide biennial breast screening program by mammography was implemented for females aged 50–69 years since 2004. In 2009, the phase II screening program has been extended to females aged 45–69 years and further expanded in 2010 to women aged 40–44 years who have a second degree relative with breast cancer in the phase III program [28]. The phase III screening program has been continuously conducted ever since 2010. Therefore, a fixed starting date of follow-up after 2009 would be less impacted by the sequential changes in the different waves of screening programs. Furthermore, we have considered the “potential detection bias” in our modeling (Table 1). The presence of “benign breast conditions” may lead to detection bias and use of estrogen may be an important risk factor of breast cancer [29]. These had not been considered in previous studies, but we have carefully addressed these potential confounders (Table 1) in our analyses.

Although the median follow-up duration of 2.8 years in our present study was relatively short, this was comparable to the median duration of pioglitazone exposure of 2.8 years in the final report of the US KPNC study (study period 1997–2012) [6] and was longer than the 1.5 years in the French study (study period 2006–2009) [5]. Because pioglitazone is not a first-line antidiabetic drug, even though the study period was longer than 10 years in the KPNC study, the median exposure time of pioglitazone was only 2.8 years [6]. It is surely justified to conduct additional studies with longer durations of follow-up or larger sample sizes to elucidate the effect of pioglitazone on breast cancer.

Based on the following additional considerations, we did not follow the patients forward from the time of drug exposure. First, pioglitazone is not a first-line antidiabetic drug and it has not been approved for clinical use in Taiwan until after 2002. If the patients were to be followed since enrollment at the time of diabetes diagnosis (from 1999 to 2008, Fig. 1) or at the time of drug exposure, the starting dates of different patients would vary remarkably throughout a long period of time and never users would surely have earlier starting dates of follow-up than ever users. This would probably introduce other unexpected bias. Second, during a long and varying starting date of follow-up, the prescription of antidiabetic drugs would be affected by the evolution of changes in treatment guidelines. Third, environmental risk factors of breast cancer and cancer diagnostic methods and screening programs should have changed at different time points of start of follow-up and these would surely introduce additional bias.

There are some clinical implications in the present study. First, together with our previous studies that do not suggest an increased risk of bladder cancer [30], ovarian cancer [31], oral cancer [32], kidney cancer [33], thyroid cancer [34], lung cancer [35] and prostate cancer [36] associated with pioglitazone use, the public health concern of an increased cancer risk associated with pioglitazone can be relieved and should not impede the clinical use of pioglitazone. Second, the potential benefits of pioglitazone on the improvement of lipid profile [37], the risk reduction of dementia [38, 39], chronic obstructive pulmonary disease [40], non-alcoholic fatty liver disease [41], stroke [2] and cardiovascular disease [42] and the usefulness of pioglitazone in the treatment of polycystic ovarian syndrome in women [43] suggest that some patients may gain pleiotropic benefits beyond glycemic control from the appropriate use of pioglitazone.

The present study has some other strengths. Because the database was derived from the whole population and they spanned the whole period from the beginning of the marketing of pioglitazone in 2002 in Taiwan [44] until the end of follow-up on December 31, 2011, the potential risk of selection bias related to sampling error could be minimized. Because the NHI covers almost the whole population of Taiwan and the database was complete and included all claim records on outpatient visits, emergency department visits and hospital admission, and we caught the diagnoses from all sources. The use of medical records would have markedly avoided self-reporting bias. Because cancer is considered a catastrophic illness by the NHI and most medical co-payments can be waived, detection bias related to different socioeconomic status might have much reduced. Furthermore, there is a low drug cost-sharing required by the NHI and patients with certain conditions such as low-income household, veterans or patients with prescription refills for chronic disease are exempted from the drug cost-sharing. The risk of detection bias would be much reduced among different social classes in Taiwan.

The study limitations included a lack of actual measurement data for potential confounders such as obesity, smoking, alcohol drinking, family history, lifestyle, dietary pattern, and genetic parameters. In addition, we did not have biochemical data such as hormonal profiles, blood glucose levels, hemoglobin A1C concentrations, insulin, C-peptide levels, or calculation of homeostasis model assessment for insulin resistance for evaluating their impacts. Another limitation is the lack of information on the pathology, grading and staging of breast cancer. Finally, we should point out that the short median follow-up time of 2.8 years and the relatively low number of breast cancer cases (Table 2: *n* = 35 in ever users in the unmatched cohort and the matched cohort and *n* = 41 in never users in the matched cohort) would potentially lead to a conclusion of null association because of lack of statistical power. Therefore, additional studies are warranted to confirm our findings.

In summary, this study supports a null association between pioglitazone use and breast cancer risk in Taiwanese female patients with type 2 diabetes mellitus. The findings of the present study together with those of our previous studies [30–36, 45, 46] should at least relieve the concern of a potentially higher risk of common cancers associated with pioglitazone use. Because of the small case numbers of breast cancer and the limited follow-up time, further studies are warranted to confirm our conclusion of a null association.

## Data Availability

Due to the law restriction of the release of the database to specific investigators for specific research aims, the database should not be distributed to unrelated persons. Requests to access the datasets should be directed to C.H. Tseng via ccktsh@ms6.hinet.net.

## Abbreviations

ICD-9-CM: International Classification of Diseases, Ninth Revision, Clinical Modification
KPNC: Kaiser Permanente Northern California
NHI: National Health Insurance

## References

[1] Dormandy JA, Charbonnel B, Eckland DJ, Erdmann E, Massi-Benedetti M, Moules IK (2005). Secondary prevention of macrovascular events in patients with type 2 diabetes in the PROactive Study (PROspective pioglitAzone Clinical Trial In macroVascular Events): a randomised controlled trial. Lancet..

[2] Kernan WN, Viscoli CM, Furie KL, Young LH, Inzucchi SE, Gorman M (2016). Pioglitazone after ischemic stroke or transient ischemic attack. N Engl J Med.

[3] Vaccaro O, Masulli M, Nicolucci A, Bonora E, Del Prato S, Maggioni AP (2017). Effects on the incidence of cardiovascular events of the addition of pioglitazone versus sulfonylureas in patients with type 2 diabetes inadequately controlled with metformin (TOSCA.IT): a randomised, multicentre trial. Lancet Diabetes Endocrinol.

[4] Ferrara A, Lewis JD, Quesenberry CP, Peng T, Strom BL, Van Den Eeden SK (2011). Cohort study of pioglitazone and cancer incidence in patients with diabetes. Diabetes Care.

[5] Neumann A, Weill A, Ricordeau P, Fagot JP, Alla F, Allemand H (2012). Pioglitazone and risk of bladder cancer among diabetic patients in France: a population-based cohort study. Diabetologia..

[6] Lewis JD, Habel LA, Quesenberry CP, Strom BL, Peng T, Hedderson MM (2015). Pioglitazone use and risk of bladder cancer and other common cancers in persons with diabetes. JAMA..

[7] Tseng CH (2014). Metformin may reduce breast cancer risk in Taiwanese women with type 2 diabetes. Breast Cancer Res Treat.

[8] Tseng CH (2017). Rosiglitazone reduces breast cancer risk in Taiwanese female patients with type 2 diabetes mellitus. Oncotarget..

[9] Tseng CH (2017). Metformin and lung cancer risk in patients with type 2 diabetes mellitus. Oncotarget..

[10] Tseng CH (2017). Metformin is associated with a lower risk of colorectal cancer in Taiwanese patients with type 2 diabetes: a retrospective cohort analysis. Diabetes Metab.

[11] Teresi RE, Shaiu CW, Chen CS, Chatterjee VK, Waite KA, Eng C (2006). Increased PTEN expression due to transcriptional activation of PPARgamma by lovastatin and rosiglitazone. Int J Cancer.

[12] Parsons LS. Performing a 1:N case-control match on propensity score. http://www2.sas.com/proceedings/sugi29/165-29.pdf (last accessed 20 Apr 2022).

[13] Austin PC, Stuart EA (2015). Moving towards best practice when using inverse probability of treatment weighting (IPTW) using the propensity score to estimate causal treatment effects in observational studies. Stat Med.

[14] Kashyap SR, Defronzo RA (2007). The insulin resistance syndrome: physiological considerations. Diab Vasc Dis Res.

[15] Tseng CH, Chong CK, Tai TY (2009). Secular trend for mortality from breast cancer and the association between diabetes and breast cancer in Taiwan between 1995 and 2006. Diabetologia.

[16] Tseng CH (2014). Diabetes and breast cancer in Taiwanese women: a detection bias?. Eur J Clin Investig.

[17] Xue F, Michels KB (2007). Diabetes, metabolic syndrome, and breast cancer: a review of the current evidence. Am J Clin Nutr.

[18] Kabat GC, Kim M, Caan BJ, Chlebowski RT, Gunter MJ, Ho GY (2009). Repeated measures of serum glucose and insulin in relation to postmenopausal breast cancer. Int J Cancer.

[19] Kotta-Loizou I, Giaginis C, Theocharis S (2012). The role of peroxisome proliferator-activated receptor-γ in breast cancer. Anti Cancer Agents Med Chem.

[20] Kocdor H, Kocdor MA, Canda T, Gurel D, Cehreli R, Yilmaz O (2009). Chemopreventive efficacies of rosiglitazone, fenretinide and their combination against rat mammary carcinogenesis. Clin Transl Oncol.

[21] Margalit O, Wang D, Dubois RN (2012). PPARγ agonists target aromatase via both PGE2 and BRCA1. Cancer Prev Res (Phila).

[22] Bojková B, Orendáš P, Kajo K, Kubatka P, Výbohová D, Bálentová S (2016). Role of high-fat diet on the effect of pioglitazone and melatonin in a rat model of breast cancer. Eur J Cancer Prev.

[23] Ece H, Cigdem E, Yuksel K, Ahmet D, Hakan E, Oktay TM (2012). Use of oral antidiabetic drugs (metformin and pioglitazone) in diabetic patients with breast cancer: how does it effect serum Hif-1 alpha and 8Ohdg levels?. Asian Pac J Cancer Prev.

[24] Nissen SE, Wolski K (2007). Effect of rosiglitazone on the risk of myocardial infarction and death from cardiovascular causes. N Engl J Med.

[25] Lewis JD, Ferrara A, Peng T, Hedderson M, Bilker WB, Quesenberry CP (2011). Risk of bladder cancer among diabetic patients treated with pioglitazone: interim report of a longitudinal cohort study. Diabetes Care.

[26] Smith RA, Saslow D, Sawyer KA, Burke W, Costanza ME, Evans WP (2003). American Cancer Society guidelines for breast cancer screening: update 2003. CA Cancer J Clin.

[27] Pivot X, Rixe O, Morere JF, Coscas Y, Cals L, Namer M (2008). Breast cancer screening in France: results of the EDIFICE survey. Int J Med Sci.

[28] Su SY (2022). Nationwide mammographic screening and breast cancer mortality in Taiwan: an interrupted time-series analysis. Breast Cancer.

[29] Chlebowski RT, Anderson GL (2012). Changing concepts: Menopausal hormone therapy and breast cancer. J Natl Cancer Inst.

[30] Tseng CH (2012). Pioglitazone and bladder cancer: a population-based study of Taiwanese. Diabetes Care.

[31] Tseng CH (2013). Pioglitazone does not affect the risk of ovarian cancer: analysis of a nationwide reimbursement database in Taiwan. Gynecol Oncol.

[32] Tseng CH (2014). Pioglitazone and oral cancer risk in patients with type 2 diabetes. Oral Oncol.

[33] Tseng CH (2014). Pioglitazone does not affect the risk of kidney cancer in patients with type 2 diabetes. Metabolism..

[34] Tseng CH (2014). Pioglitazone and thyroid cancer risk in Taiwanese patients with type 2 diabetes. J Diabetes.

[35] Tseng CH (2018). Pioglitazone and lung cancer risk in Taiwanese patients with type 2 diabetes. Diabetes Metab.

[36] Tseng CH. Pioglitazone and prostate cancer risk in Taiwanese male patients with type 2 diabetes: a retrospective cohort study. World J Mens Health. 2022. 10.5534/wjmh.210157 Epub ahead of print. PMID: 35274506. 10.5534/wjmh.210157 PMC9826906 35274506

[37] Tseng CH, Huang TS (2005). Pioglitazone with sulfonylurea: Glycemic and lipid effects in Taiwanese diabetic patients. Diabetes Res Clin Pract.

[38] Tseng CH (2018). Pioglitazone reduces dementia risk in patients with type 2 diabetes mellitus: a retrospective cohort analysis. J Clin Med.

[39] Tseng CH (2020). Dementia risk in type 2 diabetes patients: acarbose use and its joint effects with metformin and pioglitazone. Aging Dis.

[40] Tseng CH (2022). Pioglitazone and risk of chronic obstructive pulmonary disease in patients with type 2 diabetes mellitus. Int J COPD.

[41] Lian J, Fu J (2021). Pioglitazone for NAFLD patients with prediabetes or type 2 diabetes mellitus: a meta-analysis. Front Endocrinol (Lausanne).

[42] Nesti L, Tricò D, Mengozzi A, Natali A (2021). Rethinking pioglitazone as a cardioprotective agent: a new perspective on an overlooked drug. Cardiovasc Diabetol.

[43] Xu Y, Wu Y, Huang Q (2017). Comparison of the effect between pioglitazone and metformin in treating patients with PCOS: a meta-analysis. Arch Gynecol Obstet.

[44] Tseng CH (2012). Pioglitazone and bladder cancer in human studies: is it diabetes itself, diabetes drugs, flawed analyses or different ethnicities?. J Formos Med Assoc.

[45] Tseng CH, Tseng FH (2012). Peroxisome proliferator-activated receptor agonists and bladder cancer: lessons from animal studies. J Environ Sci Health C Environ Carcinog Ecotoxicol Rev..

[46] Tseng CH (2014). A review on thiazolidinediones and bladder cancer in human studies. J Environ Sci Health C Environ Carcinog Ecotoxicol Rev.

